# Comparative microarray analysis of *Rhipicephalus (Boophilus) microplus *expression profiles of larvae pre-attachment and feeding adult female stages on *Bos indicus *and *Bos taurus *cattle

**DOI:** 10.1186/1471-2164-11-437

**Published:** 2010-07-19

**Authors:** Manuel Rodriguez-Valle, Ala Lew-Tabor, Cedric Gondro, Paula Moolhuijzen, Megan Vance, Felix D Guerrero, Matthew Bellgard, Wayne Jorgensen

**Affiliations:** 1 Cooperative Research Centre for Beef Genetic Technologies, Armidale, NSW, Australia; 2 Industry Services, Queensland Primary Industries & Fisheries, Department of Employment, Economic Development and Innovation, Locked Mail Bag No. 4, Moorooka, Brisbane, QLD 4105, Australia; 3 Centre for Comparative Genomics, Murdoch University, Perth, Western Australia 6150, Australia; 4 USDA-ARS, Knipling Bushland US Livestock Insect Research Laboratory, 2700 Fredericksburg Road, Kerrville, TX 78028, USA; 5 TIGB - The Institute for Genetics and Bioinformatics, University of New England, Armidale, NSW 2351, Australia

## Abstract

**Background:**

*Rhipicephalus (Boophilus) microplus *is an obligate blood feeder which is host specific to cattle. Existing knowledge pertaining to the host or host breed effects on tick transcript expression profiles during the tick - host interaction is poor.

**Results:**

Global analysis of gene expression changes in whole *R. microplus *ticks during larval, pre-attachment and early adult stages feeding on *Bos indicus *and *Bos taurus *cattle were compared using gene expression microarray analysis. Among the 13,601 *R. microplus *transcripts from BmiGI Version 2 we identified 297 high and 17 low expressed transcripts that were significantly differentially expressed between *R. microplus *feeding on tick resistant cattle [*Bos indicus *(Brahman)] compared to *R. microplus *feeding on tick susceptible cattle [*Bos taurus *(Holstein-Friesian)] (p ≤ 0.001). These include genes encoding enzymes involved in primary metabolism, and genes related to stress, defence, cell wall modification, cellular signaling, receptor, and cuticle formation. Microarrays were validated by qRT-PCR analysis of selected transcripts using three housekeeping genes as normalization controls.

**Conclusion:**

The analysis of all tick stages under survey suggested a coordinated regulation of defence proteins, proteases and protease inhibitors to achieve successful attachment and survival of *R. microplus *on different host breeds, particularly *Bos indicus *cattle. *R. microplus *ticks demonstrate different transcript expression patterns when they encounter tick resistant and susceptible breeds of cattle. In this study we provide the first transcriptome evidence demonstrating the influence of tick resistant and susceptible cattle breeds on transcript expression patterns and the molecular physiology of ticks during host attachment and feeding.

The microarray data used in this analysis have been submitted to NCBI GEO database under accession number GSE20605 http://www.ncbi.nlm.nih.gov/geo/query/acc.cgi?acc=GSE20605.

## Background

*Rhipicephalus (Boophilus) microplus *[*R. microplus*] causes large economic losses in livestock production in subtropical and tropical regions of the world through direct effects of feeding and by transmission of significant cattle diseases, such as babesiosis and anaplasmosis -reviewed by [1-3]. The application of chemical acaricides is the conventional method for tick control however there are implicit drawbacks including the release of acaricides into the environment and the development of tick acaricide resistance [4,5]. This has prompted on-going research to develop new sustainable tick control methods [6].

Vaccination has become a potentially effective alternative for controlling tick and tick-borne diseases as demonstrated by the commercial vaccines (Gavac^® ^and TickGard^®^) derived from the Bm86 antigen of *R. microplus *[7,8]. The development of new tick vaccines with greater protection than the Bm86 derivatives has to date been slow due to the limited number of suitable target antigens identified [9]. The development of novel tick control strategies requires enhanced knowledge about the proteins expressed by different *R. microplus *stages during development, in particular during tick interactions with the host.

There are approximately 870 tick species [10], divided into three families: *Ixodidae *(683 species), *Argasidae *(183 species) and *Nuttalliellidae *(1 species). The tick life cycle occurs in two phases with the first phase entirely independent of the host where eggs hatch into larvae. The second phase involves host attachment of larvae, nymph and adult (male and female) stages. In one host, ticks such as *Boophilus *spp., all 3 instars remain on the bovine host. Consequently, the larvae of these species are the key stage for host finding, host recognition, attachment and initiation of feeding. All tick species are obligate blood feeders and female adult ticks need to ingest large amounts of blood to produce eggs to oviposit and continue the life cycle [11,12]. The recent rapid development of genomic technologies is having an impact on tick - host interaction research and can help to identify potential antigens for tick vaccine development. The availability of tick genomic resources and the current status of these technologies were recently reviewed [13]. A *R. microplus *EST database was assembled from over 42,000 expressed sequence tags (ESTs) into the gene index BmiGI, [http://compbio.dfci.harvard.edu/tgi/cgi-bin/tgi/gimain.pl?gudb=b_microplus; [14-16]. This gene index consists of 13, 643 unique transcripts derived from various tick life stages and tick strains exposed to various environmental conditions. There are also databases of cDNA sequences for *Haemaphysalis longicornis *[17] and the salivary gland of *Amblyomma variegatum *[18]. Targeted EST collections have been obtained from salivary gland cDNA libraries from *Ixodes pacificus *[19] and *I. scapularis *[20]. The first draft of the *I. scapularis *genome became available early 2008 and will be the first available complete tick genome sequence [21].

Engorgement is the most important phase of tick parasitism and the initial steps are critical to success. Tick host feeding behaviour has four stages: tick-host finding behaviour, contact and host identification, attachment site selection, and attachment and feeding [22,23]. *R. microplus *has only one host and must survive the consistent pressure of the host immune response; hence the study of proteins and genes expressed by *R. microplus *during its early feeding stages is important in developing new and efficient control methods against this ectoparasite. Recent reports describe the identification of proteins expressed during the tick attachment process [24] which have contributed to the assembly of a two dimensional (2D) database of expressed larval proteins. Similarly, a proteomic analysis of *I. scapularis *nymphs after 24 hours of host feeding was reported by Narasimhan [25]. Further reports have been published describing the molecular changes in the sialomes of *R. microplus, I. scapularis *and *I. ricinus *[22,26], and from different tick feeding instars and/or from salivary glands [2,27]. All of these reports have contributed to the knowledge of the repertoire of molecules produced by ticks and have assisted in the identification of proteins which elicit a host immune response, or proteins related to pathogen transmission such as Lyme disease [26] and babesiosis [9].

Comprehensive understanding of the molecular mechanisms which regulate the initial process of tick feeding would be incomplete if we excluded environmental effects on tick host selection and the impact of the host breed in tick genome expression. Tick resistance in cattle is generally tested by placing a standard number of larval ticks on animals and subsequently counting the number of ticks that reach maturity. *Bos indicus *(Brahman) cattle appear to develop a strong natural tick resistance whereas many *Bos taurus *breeds such as Holstein-Friesians do not [28-30]. Studies to determine the environmental influences on gene expression pattern during the tick/host interaction on such divergent hosts have not yet been addressed.

In this study, the *R. microplus *gene expression microarray [14,15] was used to compare the influence of tick resistant and susceptible bovine breeds, *B. indicus *[Brahman] and *B. taurus *[Holstein-Friesian], respectively, on the transcriptomes of *R. microplus *larvae and adult females. We report that unfed larvae have elevated expression of 47 transcripts compared to larvae exposed to *B. taurus *host for 5 hours but not allowed to feed ('frustrated larvae') and adult ticks feeding on *B. indicus *cattle have elevated expression of 43 transcripts compared to adult ticks feeding on *B. taurus*. Finally, a global analysis of the gene expression microarray of unfed larvae, frustrated larvae and adult ticks of *R. microplus *demonstrates that a total of 297 ESTs are highly expressed in the early stages of *R. microplus *feeding on *B. indicus *cattle (Brahman) compared with ticks feeding on *B. taurus *(Holstein-Friesian). This is the first tick functional genomic study providing new insights to describe the complex interaction of host specificity and host breed effects on *R. microplus *gene expression.

## Results and Discussion

### 1-Assessment of gene expression on primary stages of unfed and frustrated larvae

#### a) Transcripts highly expressed B-FL and H-FL versus unattached/unfed larvae

**Figure 1:**
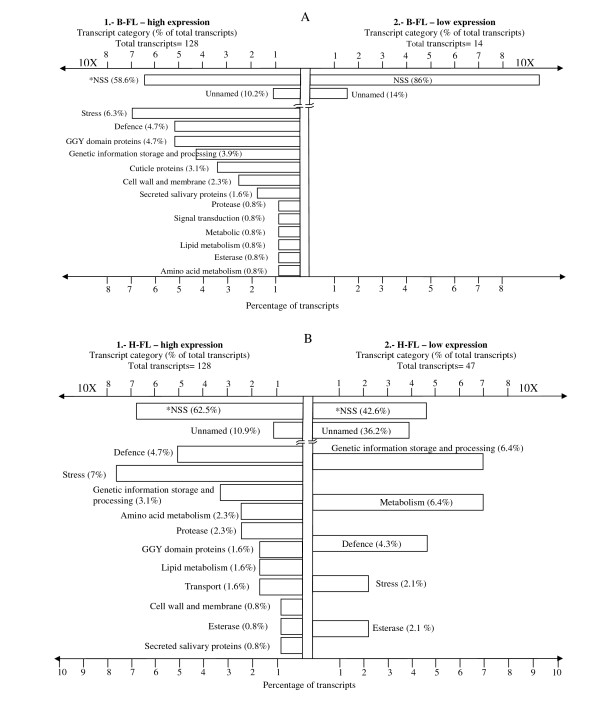
Gene expression profiles obtained by microarray comparison between *R. microplus *unfed larvae (L) versus frustrated larvae feeding on Brahman (A) and Holstein-Friesian (B) cattle.*NSS: No significant similarities. http://www.ncbi.nlm.nih.gov/geo/query/acc.cgi?acc=GSE20605.

The tick attachment and feeding process involves sequential behavioural and molecular changes which can be examined both during host recognition of newly hatched larvae and during larval host attachment while responding to feeding stimuli following host recognition. In this microarray comparison, unattached/unfed larvae (without the host stimuli [L]) versus frustrated larvae obtained after 5 hrs of exposure to *Bos indicus *(Brahman, B-FL) and *B. taurus *(Holstein-Friesian, H-FL) cattle resulted in the identification of 128 transcripts highly expressed in frustrated larvae on Brahman and Holstein-Friesian (Figure 1A.1, 1B.1). The clusters of transcripts low expressed in unattached/unfed larvae were similar in these microarray comparisons with differences only in the number of transcripts per protein family group (Figure 1A.1, 1B.1).

In both experimental groups of frustrated larvae there were highly expressed transcripts associated with GGY domain proteins. For example, H-FL had two transcripts related to GGY proteins and B-FL had a total of six transcripts, all with significant e-values (Figure 1A.1-1B.1, Table 1; Additional files 1A and 1B). GGY proteins are associated with the tick cement proteins and/or with antimicrobial activity [23,31]. However, the presence of GGY transcripts at the early stages of *R. microplus *seeking a host (frustrated larvae) suggests an important role in the process of tick-host attachment. Interestingly, all of the highly expressed *R. microplus *GGY domain transcripts had the same protein hit (TC5802, TC5872,TC6326, TC9278) at varying degrees of similarity, and the two transcripts identified in frustrated larvae on Holstein-Friesian were also highly expressed among the ten GGY related ESTs from frustrated larvae on Brahman cattle. Microbial defence transcripts were similar in B-FL (n = 6) and H-FL (n = 6) (Figure 1A.1, 1B.1, Table 1). In B-FL these transcripts included a putative microplusin, lipocalins (n = 2), female histamine binding proteins (n = 3) and only one transcript member of the protease category, metis-3 (Additional file 1A). Defence transcripts in H-FL samples were a microplusin, a lospin 1 related to lospins from *Amblyomma americanum *[32], lipocalins (n = 2), a plancitoxin-1 that has been associated with DNAse II activity [33], and an Ixoderin which is similar to the lectin Dorin M [34,35]. The protease category in H-FL was represented by three transcripts, carboxypeptidase, serine proteinase and a metalloprotease - metis 3-which is also highly expressed in B-FL (Additional file 1B).

**Table 1:** A summary of EST descriptions and the number of transcripts involved in tick attachment and host response modulation by ticks expressed differentially (p ≤ 0.001) in microarray comparisons between larvae vs frustrated larvae.

No. of *R. microplus *transcripts per category for: Larvae vs frustrated larvae microarray comparison					
	**Highly expressed in FL (Fold range)**		**Highly expressed in L (Fold range)**		

***Transcript Category***	***Brahman cattle***	***Holstein-Friesian cattle***	***Brahman cattle***	***Holstein-Friesian cattle***	***References***

Serpin-1				1 (2.5)	[50-54]
Serpin-2				1 (2.4)	[50-54]
Hemelipoglycoprotein	2 (1.6 - 2.3)	1 (1.7)		1 (1.8)	[48,61]
Esterases	1 (2)	1 (1.7)		1 (1.6)	[55-58]
Histamine Binding proteins	3 (1.8 - 2.2)				[62,63]
Lipocalin	2 (2.3 - 2.9)	2 (1.6 -2.2)			[26,38]
GGY Domain	6 (1.8 - 4.5)	2 (1.7 -2)			[23,31]
Secreted Salivary	2 (1.6 - 2)	1 (1.7)			
Cuticle proteins	4 (1.5 - 2.7)				[64,82]
Metis (metalloprotease)	1 (3)	1 (3.8)			[44-46]
Microplusin	1 (2.4)	1 (2.8)			[36,37]
Ixoderin		1 (1.8)			[43]
lospin		1 (2)			[32,41]
Serine protease		1 (1.8)			[32,41,50,68]
Carboxipeptidase A		1 (1.7)			
Cytochrome P450		1 (1.9)			
Cytochrome C oxidase	2 (1.5 -2)				[48]
Heat Shock protein	3 (1.7 - 2.5)	6 (1.6 - 3)			[47]
Glutathione S transferase	1 (1.8)				[49]
No Significant Similarities	75 (1.6 - 5)	80 (1.5 - 4.5)			
Unnamed	13 (1.7 - 2.7)	14 (1.54 - 3.2)			
**Total**	**116**	**114**		**4**	

Data set access link: http://www.ncbi.nlm.nih.gov/geo/query/acc.cgi?acc=GSE20605.

Microplusin, highly expressed in B-FL and H-FL, is an antimicrobial peptide related to tick immunity commonly found in sialotranscriptomes of related blood-feeding arthropods [36,37] (Additional files 1A and 1B). Lipocalins expressed in both frustrated larval samples are commonly associated with the modulation of the immune responses, regulation of cell homeostasis and in the clearance of endogenous and exogenous compounds. Also, they play roles in retinol and pheromone transport, olfaction, invertebrate coloration and prostaglandin synthesis. Therefore, lipocalins can perform a number of functions during tick-host interactions as they bind small molecules, interact with membrane receptors or form macromolecular complexes by binding to soluble proteins [38]. Differential expression for transcripts encoding for tick histamine binding proteins was not evident in the H-FL samples. Conversely, serine protease inhibitors (serpins) were differentially expressed in H-FL but not in B-FL (Additional files 1A and 1B). A lospin ('lone star tick serpins') transcript was highly expressed in H-FL. It is related to the 17 serpins or lospins from *A. americanum *expressed ubiquitously in the midgut, salivary glands and ovaries with lospins -1, -2, and -3 expressed at higher levels in the midgut [32,39]. These inhibitors, found in egg and larval stages of *R. microplus*, demonstrate moderate inhibition of blood coagulation enzymes, (reviewed by [40]). Furthermore, *R. microplus *serine proteinase inhibitors BmTIs from larvae have been reported as active inhibitors of trypsin, neutrophil elastase, plasmin and plasma kallikrein [40,41]. These inhibitors have a role in the modulation of the proteolytic activity identified in *R. microplus *larval life stages. The induction of protease inhibitor transcript was statistically significant in *R. microplus *larvae attaching to the *B. taurus *host indicating a host breed influence on these genes [42].

Ixoderins highly expressed in H-FL (Additional file 1B) are lectin proteins primarily expressed in tick haemocytes and salivary glands. Lectins are an essential part of non-self recognition, haemaglutination and in the transmission of pathogens in both soft and hard ticks [43]. Metis 3 transcripts were identified in both B-FL and H-FL (Additional files 1A and 1B) and members of this metalloprotease family are involved with blood meal digestion. Metis proteins are expressed by salivary glands during adult stage blood feeding in *H. longicornis *female ticks (Additional files 1A and 1B) [44] and also in the *I. scapularis *male tick [45,46].

A group of putative secreted salivary gland proteins were highly expressed similarly in both experimental groups compared to unfed larvae (B-FL n = 2, H-FL n = 1) (Table 1, Figure 1A.1-1B.1; Additional files 1A and 1B). In B-FL there were three highly expressed transcripts compared with larvae that were associated with Heat shock proteins (HSP) with another five transcripts identified as hemelipoglycoproteins (n = 2), cytochrome c oxidases (n = 2) and one glutathione S-transferase (GST) transcript (Additional file 1A). HSP transcripts (n = 6), a hemelipoglycoprotein and cytochrome P450 were highly expressed in H-FL (Table 1, Additional file 1B). HSPs are required to stabilize proteins when temperature changes from approximately 20° C to 38° C occur as the tick is ingesting warm host blood or when the tick is in contact with the skin of the warm-blooded bovine host [47]. Hemelipoglycoproteins and cytochrome c are involved in the detoxification process or energy production of larval stages [48]. The major functions of GSTs include the detoxification of xenobiotics, digestive processes, and prostaglandin synthesis and they are also associated with a series of reactions essential to protect cell constituents from oxidative attack by oxygen and oxygen-associated free radicals [49]. However, further work is needed to understand the physiological role of GSTs in tick metabolism as well as to evaluate its function during tick-host interaction.

#### b) High expressed ESTs in unfed larvae (L) *versus *B-FL and H-FL

A comparison between unfed *R. microplus *larvae versus B-FL and H-FL was conducted. Gene expression comparison between L and B-FL revealed a different transcriptome pattern compared with the differences described below for H-FL. There were only 14 transcripts highly expressed in L compared to B-FL (Figure 1A.2, Additional file 1C-1.1). All of these highly expressed transcripts had no significant similarities to known proteins (Additional file 1C-1.1).

A total of 47 genes had an elevated expression in L compared to H-FL (Table 2, Figure 1B.2, Additional file 1C-1.2) Seventeen transcripts had hits to unnamed proteins and twenty had no significant similarities to other proteins. Transcripts for serpin-1 and serpin-2 cluster in the defence group (Additional file 1A-1.2) and were highly expressed by larvae not feeding or sensing a host (L). Serine protease inhibitors or serpins are members of a ubiquitous superfamily, which target proteases, causing irreversible losses in the enzymatic activity by distorting the protease structure. Serine proteinase inhibitors are present in the arthropod haemolymph to protect their hosts from pathogens or parasites by inhibiting fungal or bacterial proteinases [50]. Also, the serine protease inhibitor gene family is involved in the regulation of several physiological functions such as the blood clotting cascade, clot resolution, the inflammatory response and complement activation [51,52], hence serine protease inhibitors are important factors which disrupt defensive host processes. Therefore, serpins play important roles prior to tick attachment as previously described [53,54,50]. However, serpins in resting larvae (L) may also function to defend against potential pathogens.

**Table 2:** Summary of EST descriptions and the number of transcripts involved in host response modulation by ticks expressed differentially (p ≤ 0.001) in microarray comparisons between Brahman frustrated larvae vs. Holstein-Friesian frustrated larvae.

*R. microplus *genes expression microarray comparison: Brahman frustrated larvae vs. Holstein-Friesian frustrated larvae		
***Transcript Category***	***Highly expressed (Fold change)***	***References***

GGY domain proteins	3 (1.3 - 2.1)	[23,31]
Serpin 2	1 (1.3)	[50-54]
Lipocalin	1 (1.3)	[26,38]
Histamine binding proteins	5 (1.2 - 1.4)	[26,62,63]
Hemelipoglycoproteins	2 (1.5 -2)	[22]
Cuticle proteins	3 (1.2 -2)	[64,82]
No Significant Similarities	19 (1.1 - 1.6)	
Unnamed	6 (1.1 -1.6)	
**Total**	**40**	

Esterases are highly variable and multifunctional hydrolytic enzymes. In insects, these enzymes are involved with various physiological activities such as regulation of juvenile hormone levels [55], digestive processes, reproductive behaviour, functioning of the nervous system, metabolism and sequestration of pesticides [56-58]. There was only one member of the esterase family in this comparison identified at a significant e-value. The role of this enzyme at this particular stage is involved in digestive processes and optimal functioning of the nervous system during early tick development stages [56-58] (Additional file 1C-1.2).

The transcript corresponding to TC12462 was identified as hemelipoglycoprotein with high similarity to vitellogenin. Tick vitellogenin sequesters heme and transfers the heme to eggs [59]. The heme biosynthesis pathway is absent in ticks, therefore they are obligate blood-feeders. Vitellogenin is an unusual heme-binding protein which is critical to embryo development because it provides ticks access to heme in the absence of a host. Hence, the biological function of the vitellogenin present in this tick instar as a storage protein related to the maturation process [59]. The remaining transcripts were genes from different metabolic pathways and genetic information storage processes.

These results suggest that some important metabolic activity in these 'resting unstimulated' larvae changes upon sensing the host. Low expressed genes were observed in frustrated larvae within the first 5 hours of larval contact with the host. Possibly, this provides preliminary evidence of gene expression regulation mediated by tick adaptation to the new environment whereby certain metabolic activities required for sustaining the larvae in the absence of the host need first to be down regulated. Perhaps if left for longer than 5 hours, these host stimulated larvae would have started to show evidence of a new gene expression pattern associated with host recognition and attachment. It was recently reported that a 24 h period of larval 'frustration' demonstrated a high metabolic activity compared to the resting unstimulated larvae [2]. The down regulation of several genes in frustrated larvae after 5 hours of *B. taurus *host exposure was recently corroborated in a parallel study undertaken within our group demonstrating a decrease in number of identified miRNAs also at this stage (Barrero *et al*., unpublished). Evidence of similar changes but at protein level was observed in the protein expression patterns induced by the host-parasite interaction in adult *R. appendiculatus *ticks that were physically detached and reattached onto the host [22,60].

Feeding behaviour has been characterized in different tick species and it is regulated by two main biological factors - the nature of the sensory input obtained by a larvae and the central nervous system processing of that input. The localization and morphology of most of the sensory receptors involved in feeding behaviour and the ultrastructures of tick neurons has been studied, but not at the transcriptome level [11,12,61]. The difference in the gene expression patterns between *B. taurus *and *B. indicus *frustrated larvae compared to unfed larvae could be a result of differences in host specific stimuli during the larval attachment phase on these breeds. In summary, transcripts differentially expressed in the unstimulated larvae [L] were largely related to important metabolic pathways or transport mechanisms necessary for sustaining the ectoparasite's physiological resting state. The role of some transcripts in unfed larval stages such as serpins remains to be studied.

#### c) Highly expressed ESTs in B-FL *versus *H-FL

**Figure 2:**
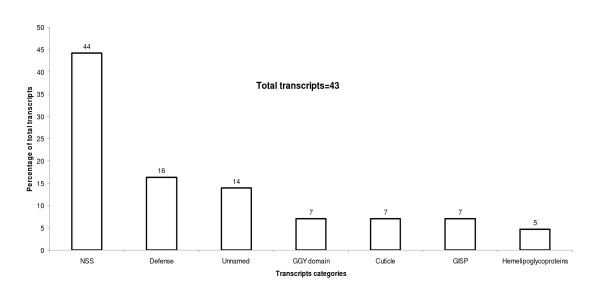
**Gene expression profile of highly expressed transcripts in the B-FL versus H-FL microarray comparison (p value ≤ 0.001)**. *NSS: No significant similarities, GISP: Genetic information storage and processing.

A comparison of frustrated larvae exposed to Brahman (B-FL) versus frustrated larvae exposed to Holstein (H-FL) demonstrated elevated expression of 43 transcripts in B-FL (Table 2, Figure 2; Additional file 1C-1.3). The majority of these high expressed genes were transcripts with no significant similarities and unnamed proteins (n = 19, 44.2% of total transcripts; and n = 6, 4% of all transcripts, respectively); three transcripts were associated with GGY proteins, TC6059; TC5802; TC9278 at e-values ranging from 1.0x10^-10 ^to 2.0x10^-07^, respectively. Additional transcripts were members of the lipocalin family similar to the *R. appendiculatus *histamine binding proteins (n = 6) and a serine protease inhibitor, serpin 2, all at highly significant e-values (Additional file 1C-1.3). Histamine is released from mast cells and basophils, often but not always mediated via an IgE-dependent mechanism, and is also released by the platelets of many mammals. Additionally, histamine is a regulator of T cell responses where binding to the lymphocyte H1 receptor results in a positive Th1 response while binding to H2 receptor results in inhibition of Th1 and Th2 responses [62,63]. Therefore, histamine binding proteins have an important role in the manipulation of host immune response together with a protease inhibitor such as serpin 2 that acts against host clotting systems and protease from the complement system [41]. It is feasible that some serpins are associated with specific tick histamine binding proteins and hence are over expressed during their activities, for instance when larvae are responding to Brahman cattle. Serpins comprise a large gene family in ticks, but the differential expression of different serpins in H-FL and B-FL do suggest different functions. However, the significance of this observation remains to be confirmed. The identification of highly expressed transcripts related to the lipocalin family - as histamine binding proteins-in larval *R. microplus *differs from results obtained by Chmelař with *I. ricinus *where transcripts for the lipocalin family were mainly detected during the later phases of adult feeding. Only one *I. ricinus *transcript was identified after 24 h of host feeding. The others were identified at 4 and 7 days of host feeding and no transcripts from the lipocalin family were differentially expressed in unfed larvae [26]. The difference in lipocalin expression pattern could be explained by the fact that *R. microplus *is a one host tick where the tick is exposed to the same host for a prolonged period of time during its whole life cycle. In contrast, *I. ricinus *is a three host tick with its larval stages feeding on small reptiles, mammals, and birds. The adult ticks feed only on large mammals, including cattle, sheep, and deer. Thus *R. microplus *may need to apply similar strategies in both the larvae and adult stages in order to combat host immune responses, which would differ from other tick species that parasitise multiple hosts.

Other transcripts differentially expressed in B-FL compared to H-FL were similar to the *Dermacentor variabilis *hemelipoglycoprotein (n = 2) with highly significant e-values (Figure 2; Additional file 1C-1.3). Cuticle protein transcripts (n = 3) were only highly expressed in B-FL and not H-FL. Cuticle proteins are a very important family particularly for immature stages of endopterygote insects which increase gradually in size during an instar [64]. In this comparison cuticle transcripts with an elevated expression in B-FL were found to be similar to *Tachypleus tridentatus *(Japanese horseshoe crab) and *I. ricinus *(sheep tick) cuticle sequences. The remaining transcripts were mostly genes associated with metabolic processing (Table 2, Figure 2; Additional file 1C-1.3).

### 2- Microarray comparison of the differential expressed transcripts in *R. microplus *feeding on Brahman versus Holstein-Friesian

The main objective of this study was to compare gene expression of *R. microplus *larvae exposed to tick susceptible and resistant breeds of cattle. The analysis of changes in gene expression in all *R. microplus *stages revealed that *R. microplus *ticks feeding on and sensing tick resistant Brahman (*B. indicus*) have a total of 297 transcripts highly expressed (p-values ≤ 0.001) (approximately 2.2% of the total 13,601 genes screened) compared with *R. microplus *ticks feeding on tick susceptible Holstein-Friesian cattle (*B. taurus*) (Additional file 2, http://www.ncbi.nlm.nih.gov/geo/query/acc.cgi?acc=GSE20605. This result confirms that significant changes occur to the *R. microplus *transcriptome when *R. microplus *ticks are confronted by different bovine host breeds. Other papers have addressed the field of vector-host interaction by the analysis of salivary gland transcripts and proteomes but our study is the first investigation which addresses how the host breed can influence the tick transcriptome [22,27]. This global comparison analysis took into account all transcripts differentially expressed in all tick stages under survey (larvae, frustrated larvae and feeding adult female tick). This analysis showed that ticks feeding on *B. indicus *(Brahman) exhibited in general a high expression of genes associated with amino acid (n = 4), lipid (n = 1), nucleotide metabolism (n = 1), general metabolism (n = 2), signal transduction (n = 1), and genes related with genetic information storage and processing (n = 7), exemplifying a high level of physiological activity of *R. microplus *tick attached and feeding on the *B. indicus *host. The majority of the high expressed transcripts represented in this comparison were of unknown function (n = 17) and no significant similarities (n = 188). The higher physiological activity of ticks feeding on Brahman cattle was also evidenced by the identification of transcripts high expressed that were: member of transport pathways (n = 4), and cell wall and membrane (n = 8) such as acetylcholinesterase, and a vitellogenin receptor (Additional file 2).

*R. microplus *has developed various mechanisms to modulate their host's haemostatic and immune defences as part of its adaptation to a blood feeding environment [36]. Ticks have developed an arsenal of molecular strategies to overcome these haemostatic mechanisms and immune defences. In our survey, 35 transcripts from defence and protease categories involved in modulating the host defences, represented 7% of the total transcripts expressed at high levels in ticks feeding on *B. indicus *cattle (Table 3, Additional file 2). For example, thrombin inhibitors (n = 3) were previously reported in the saliva of *R. microplus *as: BmAP which blocks the thrombin active site [65], microphilin which interacts with thrombin exosite I [66], and more recently, another thrombin inhibitor was identified specific to *R. microplus *gut which could assist to keep the blood from clotting during digestion [67]. A slight increase in TC9527 gut expression was determined using qRT-PCR (not shown). A transcript similar to lospin 7 (lone star tick serpin 7) could be important in the evasion of host defences. Lospins/serpin are ubiquitously expressed in the midgut, ovary and salivary glands and have been reported in *A. americanum *[32] and *I. scapularis *[68]. Another two transcripts were identified as cystatins which are reversible inhibitors of papain-like cysteine proteases only recently discovered in ticks [69]. This inhibitor family is subdivided into three closely related subfamilies: family 1, family 2 and family 3. Among them, only family 1 cystatins are intracellular. The physiological function of these proteins has been proposed to be the regulation of protein turnover and defence against pathogens as well as producing a balance in the host-parasite immune relationship [31]. In *R. microplus *only cystatin family 1, named Bmcystatin has been biochemically characterized and its function could be related to the prevention of premature degradation of vitellin [70]. The sialostatin L (cystatin) from *I. scapularis *was present in saliva and actively affected host proteolytic activity at sites of infestation. This protein also displayed an anti-inflammatory role and the inhibition of cytotoxic T lymphocyte proliferation in the host, thus contributing to the successful feeding of the tick [71].

**Table 3:** Summary of EST descriptions and number of transcripts involved in attachment and host response modulation by ticks expressed differentially (p ≤ 0.001) in the global comparison of all *R. microplus *stages attaching and feeding on Brahman vs Holstein-Friesian cattle.

Global comparison of all *R. microplus *stages attaching and feeding on Brahman vs Holstein-Friesian cattle (fold change)			
***Transcript Category***	***Highly expressed***	***Low expressed***	***References***

Histamine binding protein	5 (3 - 7)		[26,62,63]
Immunoglobulin G binding	5 (4 - 5)		[46,77]
Cystatin	2 (4)		[69,70]
Thrombin inhibitor	3 (3)		[65-67]
Lipocalin	1 (3)		[26,38]
Lospin 7	1 (3)		[32,68]
Proteinases	16 (2 - 4)		[22,53,54]
P27	12 (3 - 6)		[24,27,80]
Cuticle proteins	4 (3 - 4)		[64,82]
Secreted salivary proteins	8 (2 -6)		[22]
Glutathione S-transferase	1 (5)		[49]
Cytochrome P450	1 (3)		
Acetylcholinesterase	1 (2)		
Unnamed	17 (3 -4)	16 (2.4 - 3.9)	
No Significant Similarities	188 (2 - 6)		
**Total**	**265**	**16**	

Histamine, a principal mediator of inflammatory reactions, is released by the host in response to tissue damage. It is mainly secreted by mast cells and basophils and binds to H1 and/or H2 receptors on the surface of target cells increasing the permeability of post-capillary blood vessels allowing wound repair factors to pass into the co-damaged tissues. Blood sucking arthropods have adopted a different strategy to control histamine salivary gland extracts, with *Rhipicephalus *species of *ixodid *ticks showing an uncharacterized "histamine-blocking" activity [72]. Also the one host tick *R. microplus *remains feeding on the host prolonging its exposure to host defences, including inflammatory and immune responses. In resistant animals, rejection of ticks is often based on cutaneous hypersensitivity reactions [73]. Acquired resistance to ticks can be reduced by the *in vivo *administration of synthetic H1 and H2 receptor antagonists, implicating histamine in this rejection process [74]. In blood sucking arthropods, many lipocalin related sequences, expressed in the salivary glands, have been identified. Several have been characterized, notably RaHBP2 (*R. appendiculatus *Histamine Binding Protein 2). TC11485 (THBP-2) was confirmed as highly expressed in qRT-PCR analyses in the female salivary gland (not shown). The lipocalin proteins are implicated in the completion of the blood meal, interfering with platelet aggregation, blood coagulation, activation of the complement system and inflammation [75]. In tick feeding on *B. indicus*, transcripts associated with lipocalin or specifically with histamine binding proteins (n = 6) have an elevated expression compared with ticks on *B. taurus*. The differences in expressed genes in this comparison could be associated with the necessity for *R. microplus *to overcome a stronger innate immune response observed in *B. indicus *compared to *B. taurus *cattle [28,76].

**Figure 3:**
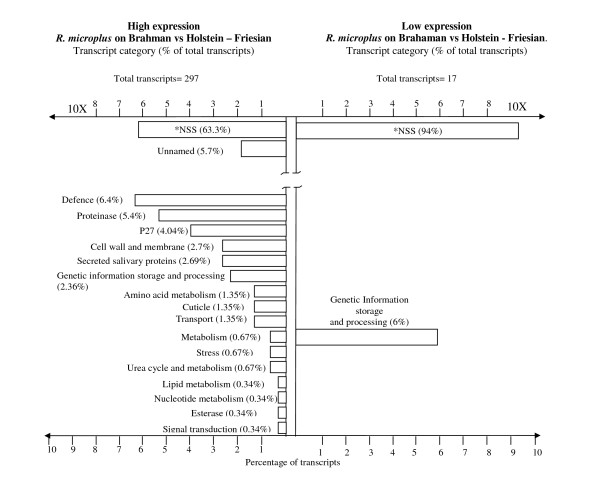
**Gene expression pattern after a Global analysis of *R. microplus *transcripts in all tick stages during attachment and feeding on Brahman cattle compared to *R. microplus *feeding on Holstein - Friesian**. *NSS: No significant similarities. http://www.ncbi.nlm.nih.gov/geo/query/acc.cgi?acc=GSE20605

Transcripts related with immunoglobulin binding (n = 5) were highly expressed in ticks feeding on *B. indicus *cattle (Table 3, Figure 3; Additional file 2) compared with the unfed larvae and the frustrated larval stages on the same host. Immunoglobulin-binding proteins are present in the tick haemolymph and salivary glands of *R. appendiculatus*, *A. variegatum*, *I. hexagonus *and *I. ricinus *and it is thought that these proteins are responsible for host immunoglobulin-G excretion via salivation, during the parasite feeding [46,77].

There is a significant increase in protease genes expressed by ticks on *B. indicus *cattle (n = 16) which is probably influenced by the tick's need to penetrate the host skin in order to reach the blood vessels. Perhaps these proteins are stimulated in a host specific manner, as their expression was absent in the same stage ticks sampled in this study from *B. taurus *breeds. Proteases can damage the capillaries and small vessels causing a bleed, which will increase the blood volume for feeding. Thus, proteases directly interfere with the blood clotting and the platelet aggregation systems of their hosts by the action of these proteases and protease inhibitors. These enzymes are probably responsible for promoting a pre-oral or even an oral digestion, acting also in the maintenance of blood in its liquid form [53]. Also, the high expressed protease transcripts could be related to degradation of collagen which is over expressed in *B indicus *with respect to *B taurus *cattle [28]. This phenomenon contributes to the efficiency of the tick feeding process that will be completed by the gut enzymes. In this context, metalloproteases are emerging as substances with toxic effects that disturb the haemostatic system and degrade the cellular matrix proteins of the host [45,78,79]. Ticks feeding on *B. indicus *had five transcripts identified as metalloproteases at high levels of significance as highly expressed in *B. indicus *compared to *B. taurus *ticks. TC6945 was confirmed as highly expressed in adult tick salivary gland tissue using qRT-PCR (not shown). Also, serine protease transcripts were highly expressed (n = 10) with high significance. In addition, transcripts corresponding to putative secreted salivary proteins (n = 8) had an elevated expression in *R. microplus *feeding on *B. indicus *and 12 different transcripts coding for P27 proteins which represents 4% of the total differentially expressed transcripts. P27 proteins are analogous to troponin I like proteins involved in actin regulation [24,80]. However, P27 from *Hyalomma asiaticum *is more similar to histamine binding proteins secreted in tick saliva [27] (Table 3, Figure 3; Additional file 2; http://www.ncbi.nlm.nih.gov/geo/query/acc.cgi?acc=GSE20605.

Adult females of *R. microplus *ingest large volumes of blood with the digestion of haemoglobin resulting in the increase in the formation of free radicals by stimulating lipid peroxidation through their interaction with lipid hydroperoxides which convert to highly toxic alcoxyl and peroxyl radicals. Therefore, it has been suggested that blood digestion is a source of oxidative stress for blood-feeding parasites, and several antioxidant defences and haeme detoxification mechanisms have been shown to contribute to the adaptation of these parasites to a hematophagous lifestyle [81]. In *R. microplus*, transcripts related to managing oxidative stress were identified (n = 2) including a transcript with significant similarity to a putative glutathione S-transferase [49].

The gradual increase in size during an instar is a characteristic of the immature stages of *R microplus*. Cuticular synthesis is at a high level in this arthropod and there is an increase in thickness as well as an increase in area of the cuticle. During tick feeding on Brahman cattle, there were genes differentially expressed described as cuticle proteins (n = 4) (Table 3, Figure 3; Additional file 2). All these genes are necessary for the cuticle adaptations that *R. microplus *requires for each instar stage, for instance adult females can increase in weight from 10-15 mg to 150-200 mg in less than 24 hrs. Throughout this time the procuticle undergoes rapid stretching while the epicuticle unfolds to allow the increase in volume [64,82]. A possible explanation to this difference is that ticks feeding on either host require expanding upon blood feeding therefore cuticle gene expression timing could be different for the ticks collected from the different breeds, or these genes are induced by factors related to the interactions with a specific host breed. A group of transcripts with unknown function (n = 16) and a transcript related with genetic information storage and processing were found to be expressed at low levels in Brahman compared to Holstein - Friesian ticks (Additional file 2; http://www.ncbi.nlm.nih.gov/geo/query/acc.cgi?acc=GSE20605.

Finally, in this survey a total of 297 genes were highly expressed in ticks attaching and feeding on *B. indicus *compared with ticks feeding on *B. taurus *which is 2% of the total *R. microplus *transcripts under study in the microarrays. This difference in the *R. microplus *transcriptome expression could be a result of the interaction between the host breed and all tick gustatory, olfactory chemo-receptors, chemo-mechano receptors associated with tick sense organs which are implicated in host localization and feeding [83]. It is thus not surprising to discover differences in the tick transcriptome upon encountering different breeds. However, how *B. indicus *have evolved to control tick numbers and whether this is a consequence of an intrinsic ability to perhaps manage odour stimuli is still not known.

### 3- Microarray results evaluations and relative quantitative RT-PCR

In order to validate the results observed by microarray analysis, fourteen randomly selected ESTs were evaluated with qRT-PCR analysis (Table 4). These were utilised to assess the relative abundance of transcripts in the larval and adult tick stages. The cDNA samples used for qRT-PCR were different to those prepared for the microarray survey as 'Methods'. Those selected to compare the larval samples included: histamine binding protein of 22.8 kDa [TC9672], GGY domain proteins [CK173243, CK174565, TC5872, TC6326], ATSP [TC13140], lipocalin-like protein [TC9228], unknown [TC5847], serpin-1 [CK184446] and a serpin-2 precursor [TC5931]. The global *R. microplus *gene expression microarray comparison showed that there are 297 genes with elevated expression in ticks feeding on and sensing Brahman cattle compared with ticks feeding on and sensing Holstein-Friesian cattle. The highly expressed ESTs selected for qRT-PCR evaluation of Brahman vs Holstein-Friesian ticks (adult and frustrated larvae) included: histamine binding 1 [TC9363], immunoglobulin G binding protein B [TC12051], Unknown protein [TC9814], and a putative salivary protein [TC8147]. The qRT-PCR demonstrated an increase in expression as determined by the arrays under the conditions of this study. Of the assays selected eight (~60% of assays; TC9228 and TC5872 are repeated in two different comparisons Table 4) had a high correlation with the microarray experiment at 0.88. When all assays are included in the statistical analysis a lower correlation 0.58 is observed. This could have been influenced by a number of factors including the fact that a third set of samples were used in the qRT-PCR analyses and not a pool of the original microarray samples. Furthermore, the selection of housekeeping genes for *R. microplus *expression normalization is not yet a standardised technique. Most researchers utilise a single housekeeping gene [14]; [2], though, for accuracy in quantitative gene expression analyses, it is recommended to add housekeeping genes until stable fold change is observed [84]. Stable fold change levels were not obtained in this study for all transcripts (data not shown), using three housekeeping genes for normalization. However, there are currently few known housekeeping genes available for *R. microplus *expression analysis as confirmed in a recent publication [85]. As more tick microarray data is now available, this will enable the identification of stable housekeeping genes to standardise *R. microplus *qRT-PCR in the future (manuscript under preparation).

**Table 4:** Microarray evaluation by qRT- PCR using fourteen highly expressed genes from different tick stages to confirm microarray differential expression (TC5872 and TC9228 are utilized in 2 comparisons).

ID	Description	Comparison	Microarry fold change	qRT-PCR fold change^a^
CK173243	GGY domain protein	BFL × L	4.522	0.161
TC5847	Unknown	BFL × L	2.493	6.238^b^
TC13140	ATSP homologue	BFL × L	1.856	0.304
CK174565	GGY domain protein	BFL × L	3.861	0.512
TC5872	GGY domain protein	BFL × L	2.609	0.791
TC9228	Lipocalin-like protein	BFL × L	2.876	3.888^b^
TC6326	GGY domain protein	HFL × L	1.732	2.069^b^
CK184446	Serpin-1	HFL × L	2.452	0.176
TC5872	GGY domain protein	HFL × L	2.073	0.6323
TC9228	Lipocalin-like protein	BFL × HFL	1.27	3.888^b^
TC9672	Histamine binding protein 22.8kDa	BFL × HFL	1.249	1.459^b^
TC5931	Serpin-2 precursor	BFL × HFL	1.294	1.124^b^
TC9363	Histamine binding protein-1	B × H	6.713	9.955^b^
TC12051	Immunoglobulin G binding protein B	B × H	4.446	8.748^b^
TC9814	Unknown protein	B × H	4.124	4.715^b^
TC8147	Putative salivary protein	B × H	3.016	0.484

^a^qRT-PCR was conducted with a third cDNA preparation (sample 3) of the different tick stages.

^b^These qRT-PCR assays demonstrated a high correlation at 0.88 with the microarray data (linear regression with R squared 0.78)

## Conclusion

In this study we provide the first transcriptome evidence which demonstrates differences in the transcripts expression pattern of ticks feeding on tick resistant and susceptible cattle breeds. *R. microplus *ticks express genes differentially as life stages change and some of these differences can be influenced by host breed. Larvae stimulated by the *B. indicus *host expressed a higher number of proteins involved in tick attachment such as putative cement-associated proteins (GGY domain proteins), proteases required for blood meal digestion, oxidative stress adapting proteins, and defences against the host mediators, such as anticoagulants, immunosuppressants, and histamine-binding proteins. As feeding progressed in the adult female stage, a new set of transcripts had an elevated expression on Brahman cattle including immunoglobulin binding proteins and cuticle proteins, as well as transcripts with similar functions to those highly expressed during the initial larval attachment phase. *R. microplus *ticks on resistant Brahman cattle exhibited similar patterns of gene expression as ticks on susceptible Holstein-Friesian in respect to gene categories with some changes in the number and types of transcripts differentially expressed. Many of the high and low expressed transcripts identified could not be assigned function as a fully annotated tick genome is not yet available. The qRT-PCR for evaluation of the gene expression corresponded well with the microarray gene expression analysis. This is the first study to demonstrate molecular evidence for the basis of differences in tick gene expression on tick resistant versus susceptible hosts.

## Methods

### Ticks and animal sampling

A total of six tick-naïve female cattle aged 20 months, three Brahman and three Holstein-Friesians were maintained in a set of concrete yards, free of ticks, until trial commencement. These cattle were infested with 1.5 g (~30,000) - 21 days old - N strain larvae [86] and were maintained on pasture at the University of Queensland's Pinjarra Hills campus. On Day 2, approximately 20,000 larvae were placed into a 24 cm^2 ^mesh bag to prevent feeding and attached to the neck of each animal for 5 hrs in order for the larvae to 'sense' host stimuli while also in the presence of other attached ticks. These 'frustrated' larvae from both the Brahman (B-FL) and Holstein-Friesian (H-FL) were immediately frozen in liquid nitrogen after collection for total RNA extraction. An additional 20,000 unattached larvae (unfed) kept under laboratory conditions (at 27°C and approximately 90% relative humidity) were processed to provide a control larvae group without host stimulus. At 17 days (soon after the 2^nd ^tick moult, from nymphs to adult stages) approximately 500 young adult female ticks were collected from the Brahman and Holstein-Friesian cattle and frozen immediately after collection for total RNA extraction. The sampling regime was repeated to provide biological replicates of each sample (1 and 2 denote biological replicates). The experimental samples were: unattached/unfed larvae: L1, L2. Frustrated larvae: B-FL1; B-FL2 and H-FL1; H-FL 2. Attached adult female ticks (~17 days): -B-AT1, B-AT2 and H-AT1, H-AT2. (B = Brahman; H = Holstein-Friesian; FL = frustrated larvae; AT = adult tick). This protocol was performed in accordance with guidelines approved by the University of Queensland Animal Ethics Committee (Approval No. SVS/872/07/CRC).

### Total RNA extraction

For each treatment and replicate, RNA was prepared from approximately 20,000 frustrated larvae and 500 adult ticks collected as described above. The ticks were ground in liquid nitrogen using a sterile mortar and pestle and total RNA was isolated using the TRIzol^® ^reagent according to the manufacturer's protocol (GibcoBRL, USA). The mRNA was purified from these samples using the Poly (A) Purist™ MAG Kit (AMBION, USA) as recommended by the manufacturer.

### cDNA Synthesis

cDNA was prepared from the above mRNA samples using the SuperScript™ Double-Stranded cDNA Synthesis Kit (Invitrogen, USA) as recommended by the manufacturer with the exception that after the second strand cDNA synthesis was terminated by the addition of 0.5 M EDTA and before the phenol: chloroform: isoamyl alcohol step, a RNase A treatment step was included as recommended in the NimbleGen cDNA protocol (NimbleGen, USA). After this RNase A treatment, the Superscript double stranded cDNA Synthesis protocol was followed as described in the technical manual. cDNA median size was verified by 1% agarose electrophoresis in TAE 1X, and 2 μg of each cDNA sample were sent to NimbleGen Systems Inc. (Madison, WI, USA) for microarray hybridization using the *R. microplus *custom array (NimbleGen Custom Design name: 2006-05-22_*B_microplus*_50mer_exp).

### *R. microplus *expression microarray

The microarray was prepared by NimbleGen Systems Inc. following the method reported by Saldivar [14] (GEO approved http://www.ncbi.nlm.nih.gov/geo/query/acc.cgi?acc=GSE20605. Briefly, the high-density single channel oligonucleotide arrays were constructed by NimbleGen Systems Inc. target 13,601 of the 13,643 members of BmiGI Version 2 with 14 perfect match 50-mer probes per BmiGI target. No mismatched probes were included on the arrays, although probes with randomly generated sequences are included. These random sequence probes were designed to match the melting temperature (Tm) of the other probes on the array and to reflect the distribution of non-specific signal intensities for binding events to probes with approximately the same composition as the perfect match probes but with random sequences.

Each microarray chip includes two in-slide replicates (spot replicates). In this experiment two biologμical replicates for each sample were hybridized to assess variability between individual samples. Each pooled sample consisted of twenty thousand unfed and frustrated larvae and five hundred adult ticks obtained from Brahman and Holstein - Friesian cattle as the source of RNA. Ten *R. microplus *samples were hybridized to individual microarrays at NimbleGen Systems Inc. (Madison, WI, USA).

### Statistical analysis

Following array hybridization the raw intensity values were background corrected using convolution, and normalized using quantile normalization to adjust for technical sources of variation. Final log2 expression intensities were generated using the Robust Multichip Average (RMA) algorithm [87]; [88] implemented in R.

Differential expression was tested on the RMA normalized intensities using a mixed model of the form *y_ijkr _= μ + BS_rk _+ G_i _+ GT_ij _+ E_ijkr _*where *y_ijkr _*were the log2 RMA normalized signal intensities; *i *= gene, *j *= treatment, *r *= block, *k *= slide/array, the main fixed effect is *BS_rk _*(block by slide interaction), *G_i _*is the main random effect of the gene; *GT_ij _*is the random interaction term of the gene by treatment; *E *is just the error term; and normal assumptions for the random effects - *iid *are assumed. The model was fitted using VCE4.0 [89]. Differentially expressed (DE) probes were considered as those which were three or more standard deviations away from the mean (p-value < 0.001) and these have been used in subsequent analyses in this manuscript. The main comparisons of interest were between equivalent tick samples exposed to different breeds e.g. frustrated larvae on Brahman compared to frustrated larvae on Holstein-Friesian. The putative identities of the lists of high and low expressed tick sequences (DE probes) were then subsequently analysed using bioinformatic tools to predict function (described below).

### Bioinformatics

Nimblegen tick microarray differentially expressed probes with fold changes > 1.5 and p-value < 0.001 were screened against the following databases: Grendel HPC system [90]; NCBI protein [National Centre for Biotechnology Information: http://www.ncbi.nlm.nih.gov], String [91], COG [92], tigr_bmigi.062608 [93] and NCBI Conserved Domain database [94]. All alignments were conducted using the BLAST program suite [35] except for the NCBI Conserved Domain data where RPSBLAST was used. The alignment results were then summarized using BIOPERL scripts based on alignment percent identity (PID), query coverage and expected value threshold. The expected value and description is shown for all differentially expressed blast hits in Additional files 1 and 2. For categories not found in the COG database [95], categories were assigned manually. A cutoff e value < 0.001 was utilized in BlastX analysis [14].

### Microarray result verification

The verification of array results was based on their level of differential expression and the amount of annotation available for their corresponding BmiGI V2 sequence. A third sampling of *R. microplus *ticks (L3, H-FL3, B-FL3, B-AT3 and H-AT3) was undertaken to prepare RNA/cDNA for qRT-PCR analysis to validate the microarray results. Relative quantitative reverse transcriptase-PCR (qRT-PCR) assay based on 14 differentially expressed transcripts using gene specific primers are described in Table 4 and 5. Primers for the qRT-PCR assays were designed using Emboss Version 6.0.1 eprimer3 [96] using the following parameters: -minsize 22, -osize 24, -maxsize 27, -mintm 55, -maxtm 65, -maxpolyx 4, -gcclamp 2, -productsize 100, -mingc 35, -maxgc 65. Primer sets were subsequently screened against *R. microplus *nucleotide sequences using Blastn [97] with an expected value of 100 with targeted ESTs. Primer alignments were also screened using a custom Bioperl [98] script for forward and reverse matches to ensure these sets would not amplify bovine sequences. RNA from each of the samples was prepared as described above. cDNA was synthesized using Superscript™ III First-Strand Synthesis System for qRT-PCR (Invitrogen Corp, CA, USA) and duplicate qPCRs (10 ng per reaction) undertaken using the SensiMix dT kit (Quantace Ltd, Watford, UK) in the Corbett RotorGene 3000 (QIAGEN/Corbett, Sydney, Australia) using the following profile: 95° C 10 min, 45 cycles of 95° C 15 sec, 50 or 60° C 30 sec (see Table 5 for optimal temperatures per assay), 72° C 30 sec, followed by a melt analysis 55-90° C 30 sec on the first step, 5 sec holds for subsequent steps, according to manufacturer's instructions for SYBR green detection. All assays were first optimised on a cDNA pool consisting of whole adult female, adult male and larval cDNAs prior to screening the larval and feeding adult samples prepared from *B. taurus *and *B. indicus *cattle. Assays with the observed consistent amplification of duplicates on a standard curve (R^2 ^> 0.95) giving efficiency values of 2.0 (within 15%) were considered acceptable for normalization and expression analysis. The expression profiles (average of 2 reactions) were normalized against three housekeeping genes: *R. microplus *actin gene [99], *R. microplus *18 S rRNA gene [14], and TC11706 *R. microplus *eukaryotic elongation factor homologue [100] using the normalization strategy of [84]. Each normalized expression profile was described relative to that of the control cDNA pool. Normalized values were used to calculate the fold change (on a log 2 scale) on 14 ESTs between the microarray contrasts. A simple linear regression analysis was performed to correlate fold changes calculated by qRT-PCR analysis with fold changes estimated from the microarrays using the statistical computing.

**Table 5:** Primer sequences (5' to 3') for the 14 qRT-PCR validation assays including 18 S, actin and eukaryotic elongation factor [TC11706] housekeeping primer sets utilised for normalisation

	Anneal Temp° C	Forward	Reverse
**^a^18 S AFO-18656-363**	**60**	CCTGAGAAACGGCTACCACATC	GTGCCGGGAGTGGGTAATT
**^a^TC11706**	**60**	CAAAGGACTCAAGGACTCTCTGC	ACGAGAACTGGTCAGTAAAGAAGC
**^a^Actin**	**60**	GACATCAAGGAGAAGCTYTGC	CGTTGCCGATGGTGATS
**CK173243**	**60**	ACGAGTTGACTGATTTGAATGTGG	CAGGGAGGTACTGCTAGTGTCG
**TC5847**	**60**	CAGCATTTAGTGGTATCCGTAGC	GTGGATACGGAGGAGGATATGG
**CK174565**	**60**	AGGGATACAACCACAACTCAGG	CGAAAGAAGACTGTCCGAATCC
**TC9363**	**60**	CATTTTCTGACGATAATTGCTACG	CAGCGTACTCATTGAACTTCTCC
**TC9228**	**60**	CTACTAGCTCTCGCCTTTCTCG	CAGTAAGTCTCGTACGGGAAGC
**TC13140**	**60**	CACTTTAGTTCGCAGTCTGTGG	GTTCACGATAGAGACATGAAGACG
**TC5872**	**60**	AGGGATACAACCACAACTCAGG	CGAAAGAAGACTGTCCGAATCC
**TC9672**	**60**	CGGTTCTGAGAAATTATTGAAGC	ATCTATAGTGACTGCACCACTTGC
**TC5931**	**60**	TTGTCTATCTTGAACTTGGGAAGG	CTGTCAAACAAGTTCTGACTATCG
**TC6326**	**60**	ACAGTCCTCTTTTGGAATGACG	CACTTTGGAGCATATCTGTAATGG
**CK184446**	**50**	TCAGCTTGTCTATCTTGAACTTGG	CTGTCAAACAAGTTCTGACTATCG
**TC12051**	**60**	AGGCCTATTACATGATGCTCTACC	TGTCAATTTATTGAGTTGCGTAGC
**TC9814**	**60**	TAAAGGCTGGAGTATCCGAATG	GCAAGTTTGTCAGGATTGTACG
**TC8147**	**50**	GAATTTCAGAGAGGAATCAAGTGC	GAACAGTTTCTTTCACGGAACC

^a^Housekeeping genes utilised for normalisation of qRT-PCR

## Authors' contributions

MRV contribute to the conception and design of the project, conducted cattle tick infestation experiment, purification of mRNA and cDNA sample, performed analysis and interpretation of the data and drafted the manuscript. ALT data interpretation and helped conceived the project and participated in its coordination. CG applied statistical method for Microarray Data analysis. PM Bioinformatics script of Microarray data. MV conducted PCR reactions and FDG, MB participated in the project coordination. WJ Tick infestation, critically revised the manuscript. ALT, FDG critically revised the manuscript. All authors read, helped to edit, and approved the final manuscript.

## Supplementary Material

Additional file 1 **1A - Microarray data of Transcripts low expressed in unattached/un-fed Larvae versus B-FL**. 1B - Microarray data of low expressed transcripts in unattached/unfed Larvae versus H-FL. 1C -Microarray data of high expressed transcripts in larvae vs B-FL, larvae vs H-FL and B-FL vs H-FL. 1C.1. Unfed larvae vs B-FL. 1C.2. Unfed larvae vs H-FL. 1C.3. B-FL versus H-FL Click here for file

Additional file 2 **2 - Global comparison Microarray of transcripts differentially expressed in ticks feeding on Brahman and Holstein-Friesian cattle**. 2.1. High expressed transcripts in the microarray comparison between ticks on Brahman versus Holstein-Friesian. 2.2. Low expressed transcripts in the microarray comparison between ticks on Brahman versus Holstein-Friesian Click here for file
